# Supplementary material for: The relationship between the nurses’ work environment and the quality and safe nursing care: Slovenian study using the RN4CAST questionnaire
Source: PLoS One. 2021 Dec 20;16(12):e0261466. doi: 10.1371/journal.pone.0261466 (PMC8687596; doi:10.1371/journal.pone.0261466)

*S1 Figure****:*** *Average degree of agreement (M – arithmetic mean) and standard deviation (SD) for statements relating to the perception of the work environment.*


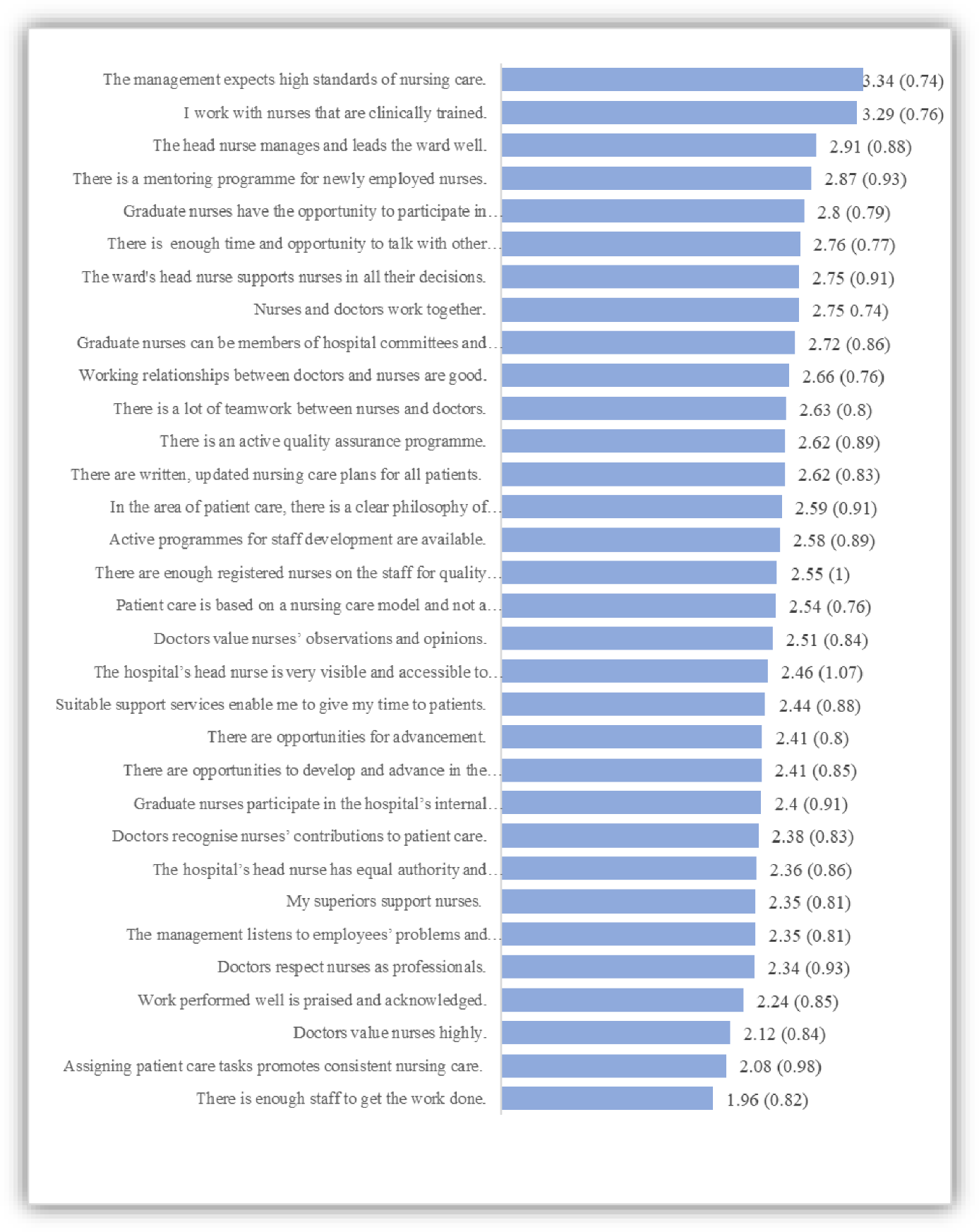

Supplement: S1 Fig — (DOCX) [file pone.0261466.s001.docx]
