# Supplementary material for: The relationship between the nurses’ work environment and the quality and safe nursing care: Slovenian study using the RN4CAST questionnaire
Source: PLoS One. 2021 Dec 20;16(12):e0261466. doi: 10.1371/journal.pone.0261466 (PMC8687596; doi:10.1371/journal.pone.0261466)
Supplement: S1 Table — (DOCX) [file pone.0261466.s002.docx]

S1 Table: Factor weights (>0.40) obtained by factor analysis (principal axis factoring method and orthogonal rotation) on statements regarding work environment.

|  | **Factor weights** | | | |
| --- | --- | --- | --- | --- |
|  | **F1** | **F2** | **F3** | **F4** |
| A_1_14 Work performed well is praised and acknowledged. |  |  |  | 0.44 |
| A_1_11.The hospital’s head nurse is very visible and accessible to the staff. |  |  |  | 0.50 |
| A_1_23 The management listens to employees’ problems and responds to them. |  |  |  | 0.51 |
| A_1_22 The department’s supervising nurse supports nurses in their decisions, even when they disagree with the doctor. |  |  |  | 0.54 |
| A_1_10 The supervising nurse manages and leads the department well. |  |  |  | 0.76 |
| A_1_31 There are written, up-to-date nursing care plans for all patients. |  | 0.45 | 0.40 |  |
| A_1_32 Assigning patient care tasks promotes consistent nursing care (i.e., a patient is cared for by the same nurse day after day). |  |  | 0.41 |  |
| A_1_27 There is a mentoring program for newly hired nurses. |  |  | 0.41 |  |
| A_1_12. There is enough staff for the work to get done. |  |  | 0.54 |  |
| A_1_19 There is a clear philosophy of nursing care in the area of patient care. |  |  | 0.59 |  |
| A_1_24. There is an active quality assurance program. |  |  | 0.64 |  |
| A_1_28 Patient care is based on a nursing care model and not a medical model. |  | 0.54 |  |  |
| A_1_4 Active employee development programs or ongoing training for nurses are available. |  | 0.56 |  |  |
| A_1_6 Registered nurses have the opportunity to participate in preparing nursing care instructions. |  | 0.57 |  |  |
| A_1_29. Registered nurses are able to be members of hospital committees and nursing care committees. |  | 0.59 |  |  |
| A_1_25 Registered nurses participate in the hospital’s internal management (e.g., on professional committees). |  | 0.63 |  |  |
| A_1_3 My superiors support nurses. | 0.54 |  |  |  |
| A_1_17 There is a lot of teamwork between nurses and doctors. | 0.67 |  |  |  |
| A_1_30 Doctors value nurses highly. | 0.72 |  |  |  |
| A_1_2 Working relationships between doctors and nurses are good. | 0.72 |  |  |  |
| A_1_13 Doctors recognize nurses’ contributions to patient care. | 0.73 |  |  |  |
| A_1_26 Nurses and doctors work well together. | 0.74 |  |  |  |
| A_1_21 Doctors respect nurses as professionals. | 0.80 |  |  |  |
| A_1_1 Suitable support services enable me to dedicate time to my patients. |  |  |  |  |
| A_1_8 I have enough time and opportunity to talk with other nurses about problems with patient care. |  |  |  |  |
| A_1_9 There are enough registered nurses on the staff for quality patient care. |  |  |  |  |
| A_1_15 The management expects high standards of nursing care. |  |  |  |  |
| A_1_16 The hospital’s head nurse has equal authority and authorization as the hospital’s top management staff. |  |  |  |  |
| A_1_18 There are opportunities for advancement. |  |  |  |  |
| A_1_20 I work with nurses that are clinically trained. |  |  |  |  |
|  |  |  |  |  |
| **The share of explained variance** | **16.4** | **10.3** | **9.1** | **8.9** |
| **Cronbach's alpha coefficient** | **0.92** | **0.82** | **0.74** | **0.76** |

**S1 Table legend:**

**F1:** Interpersonal relationships and teamwork

**F2:** Nurses' co-decision-making and development prospects

**F3:** Organisational priorities regarding the quality of patient care

**F4:** Support of the nursing management
