# Supplementary material for: The relationship between the nurses’ work environment and the quality and safe nursing care: Slovenian study using the RN4CAST questionnaire
Source: PLoS One. 2021 Dec 20;16(12):e0261466. doi: 10.1371/journal.pone.0261466 (PMC8687596; doi:10.1371/journal.pone.0261466)
Supplement: S2 Table — (DOCX) [file pone.0261466.s003.docx]

S2 Table: Factor weights (>0.40) obtained by factor analysis (principal axis factoring method and orthogonal rotation) on statements regarding patient safety culture.

| **Rotated Factor Matrix^a^** | **Distrust, poor communication**  **(F1)** | **Lack of safety culture (F2)** |
| --- | --- | --- |
|  | **Factor weights** | |
|  | **Mutual trust** | **The importance of safety culture** |
| It seems to staff that others resent their mistakes.(R) | 0.40 |  |
| When changing shifts important information about patient care is often lost.(R) | 0.83 |  |
| When moving from one unit to another things are “poorly handed over”.(R) | 0.65 |  |
| The staff openly express doubts about their superiors’ decisions and actions.(R) | 0.57 |  |
| In our unit we talk about how to make sure not to repeat mistakes. |  |  |
| We receive feedback on changes made in response to reports on events. |  | 0.72 |
| The hospital management’s actions show that patient safety is one of the most important tasks. |  | 0.77 |
| **The share of explained variance** | **23.9** | **19.7** |
| **Cronbach's alpha coefficient** | **0.72** | **0.71** |

**S2 Table legend:**

**F1**: Mutual trust

**F2**: The importance of patient safety and feedback
