# Supplementary material for: The relationship between the nurses’ work environment and the quality and safe nursing care: Slovenian study using the RN4CAST questionnaire
Source: PLoS One. 2021 Dec 20;16(12):e0261466. doi: 10.1371/journal.pone.0261466 (PMC8687596; doi:10.1371/journal.pone.0261466)
Supplement: S3 Table — (DOCX) [file pone.0261466.s004.docx]

*S3 Table: Spearman's correlation coefficient between the general assessment of the quality of care, the assessment of self-care and the assessment of the quality of care in the previous year and the perception of the work environment*

|  |  | Interpersonal relationships and teamwork | Nurses’ co-decision-making and the opportunity for development | Organisational priorities according to the quality of care | Management supports nursing care | Assessment of the work environment in your workplace in the hospital |
| --- | --- | --- | --- | --- | --- | --- |
| How would you generally assess the quality of nursing care in your department? | r | 0.12 | 0.25 | 0.32 | 0.27 | 0.32 |
|  | P | 0.160 | **0.002** | **< 0.001** | **0.001** | **< 0.001** |
| How certain are you that your patients can care for themselves after discharge? | r | 0.20 | 0.36 | 0.39 | 0.24 | 0.29 |
|  | P | **0.015** | **< 0.001** | **< 0.001** | **0.004** | **< 0.001** |
| In the past year, the quality of patient care in your hospital... | r | 0.12 | 0.29 | 0.30 | 0.34 | 0.27 |
|  | P | 0.138 | **< 0.001** | **< 0.001** | **< 0.001** | **0.001** |

r = Spearman's correlation coefficient
