# Supplementary material for: The relationship between the nurses’ work environment and the quality and safe nursing care: Slovenian study using the RN4CAST questionnaire
Source: PLoS One. 2021 Dec 20;16(12):e0261466. doi: 10.1371/journal.pone.0261466 (PMC8687596; doi:10.1371/journal.pone.0261466)
Supplement: S4 Table — (DOCX) [file pone.0261466.s005.docx]

*S4 Table: Perception of the work environment and nurses' education*

|  | A bachelor’s degree | M | SD | P |
| --- | --- | --- | --- | --- |
| **Interpersonal relationships and teamwork** | Yes | 2.45 | 0.69 | 0.562 |
|  | No | 2.51 | 0.60 |  |
| **Nurses’ co-decision-making and the opportunity for development** | Yes | 2.52 | 0.66 | 0.143 |
|  | No | 2.66 | 0.47 |  |
| **Organisational priorities regarding the quality of patient care** | Yes | **2.33** | **0.66** | **0.015** |
|  | No | **2.59** | **0.56** |  |
| **Management support nursing care** | Yes | 2.55 | 0.69 | 0.72 |
|  | No | 2.51 | 0.59 |  |
