# Supplementary material for: The relationship between the nurses’ work environment and the quality and safe nursing care: Slovenian study using the RN4CAST questionnaire
Source: PLoS One. 2021 Dec 20;16(12):e0261466. doi: 10.1371/journal.pone.0261466 (PMC8687596; doi:10.1371/journal.pone.0261466)
Supplement: Attachment — Submitted filename: Reply to reviewer 1.docx [file pone.0261466.s006.docx]

**The relationship between the nurses' work environment and the quality and safe nursing care: Slovenian study using the RN4CAST questionnaire**

*Dear reviewer,*

*thank you for* *your valuable comments and concerns which will be answered items by items below. Please find our answers which are included also into the manuscript with all necessary informations. We hope that answers we will provide will be satisfactorily to you.We believe that with your comments and our answers the clarity of the manuscript has improved.*

**The abstract provides a balanced summary of what was done and the key findings.**

*Dear reviewer**, thank you for your valuable comment.*

**The introduction could be strengthened as it does not provide a strong rationale for the study question and why it is important. The reasons for exploring the education of nurses should also be clear in the introduction. Appropriate permissions for the study appear to have been granted.**

1. *Dear reviewer, thank you for your valuable comments. Please find a text explaining the current situation in nursing in Slovenia and reasons for researching the educational differences of nurses added in the Section Introduction, where it also contains permission to use the questionnaire. These changes are shown below (underlined).*

In 2018, according to the National Institute of Public Health, only 34.7% of all nursing staff in the country (21644) had a bachelor*’*s degree (14). Analysis of data on the Slovenian categorization of the intensity of hospital nursing shows shortage of nursing staff since 2007, and the share of the deficit of Slovenian nursing staff in hospitals has been increasing over the years (in 2013 - the deficit 21.78%; in 2016 - the deficit 25.40%) (15). Analysis of data of the above-mentioned categorisation of the sample in 2011 proves that the nursing providers in Slovenian hospitals are overburdened, and there is a trend towards increasing complexity of hospital nursing (16). Dissatisfaction with work among Slovenian nurses stems mainly from the organisation of work, non-compliance with personnel standards and norms and unregulated conditions in the field of competencies between individual professional profiles (17). The International Organization of Nurses draws attention to the fact that an unsustainable heavy workload is also associated with increased absenteeism and employee turnover, which in turn compromises the quality of patient care (18). In Slovenia, nurses also change to less demanding healthcare jobs, to healthcare jobs abroad or even leave the profession.

According to the presented situation in nursing, we intended to find out how nurses perceive the current situation in their work environment. The main goal of the study was to determine whether the results confirm the correlation of the work environment to the culture of patient safety in the hospital, and consequently to the quality of care. Due to the different educated hospital nursing providers, we investigated whether the perception is influenced by the level of education achieved.

Consent to use the questionnaire for nurses from European research was obtained in August 2018 by the European coordinator of the EU-FP7 RN4CAST-project, Full Professor Walter Sermeus, provided that we conduct a study in one hospital. Therefore we decided to conduct a study among graduate nurses and healthcare technicians at the University Medical Centre Ljubljana (UMC Ljubljana). UMC Ljubljana is the largest Slovenian hospital with 2150 beds. In early 2019, 1721 nurses were employed in hospital departments of UMC Ljubljana, of which 47.3% were nurses with a bachelor*’*s degree (19).

2. *Dear reviewer, please find in Section Results/ Perception of the work environment and nurses' education a text explaining the education of nurses in Slovenia (what are the main differences among graduate nurses and healthcare technicians), as shown below.*

We then investigated whether there is a difference in the perception of the work environment according to nurses' acquired education (with a bachelor*’*s degree/ without a bachelor*’*s degree). The study involved 63.3 % graduate nurses (at the time of the study, their proportion at the hospital’s departments was 43.1%). Nurses with a bachelor*’*s degree gave a lower overall assessment of the work environment (U-value=1821.5, p=0,003). Also, nurses without a bachelor’s degree rated the dimension organizational priorities regarding the quality of patient care more positively (M=2.59, SD=0.56) than nurses with bachelor’s degrees (M=2.33, SD=0.66, p=0.015).

In Slovenia, nursing education takes place in two stages, in accordance with the standards of the European Directive 2013/55/EU. The professional competencies of the graduate nurse as a healthcare provider in the healthcare team comply with the competencies of the above-mentioned directive after completing 3-years of the first cycle of Bologna higher education. After completing four years of education, the healthcare technician is qualified to observe and monitor the patient's state of health, perform tasks as directed by a graduate nurse or medical doctor, report in the team, assist in patient activities of daily living, provide first aid, and care for the dying and deceased. His/her professional competencies enable him/her to carry out part of the nursing activities and diagnostic-therapeutic program independently, and part as a co-worker in a team led by a graduate nurse (25).

3. *Dear reviewer, please find in Section Methods/ Study and data analysis other permissions for the study, as shown below.*

Ethical approval for the pilot study was granted by the Committee for Medical Ethics of the Republic of Slovenia (Reference no. KME RS.0120-490/2018/5). Before the start of the research work, we addressed a request for a pilot study to the head nurse of UMC Ljubljana. We also previously obtained consent for research from the hospital research group in nursing and all other necessary permits (Leadership of the Division of Internal Medicine, Leadership of the Division of Surgery).

**The technique for validation of the questionnaire (principal axis factor method with orthogonal rotation) should be referenced. The validation of this tool and factor weights take up a lot of space in this paper and could be presented in supplementary material instead (Tables 1 and 2). It could be clearer how the factors were used in the analysis.**

*Reply: Dear reviewer, thank you for your valuable comments. Please find in the manuscript in Section Methods/ Measures and validation of the Slovenian version of the questionnaire inclusions of our answers, as shown below. Tables 1 and 2 are moved to The Section Supporting information (underlined). We have to say that the other reviewer also found the same issue to be discussed as you, so with this reply we answer to both of you.*

*At this point we want to mention that the validation of the questionnaire and statistical analysis is the work of statistician Vanja Erčulj, PhD, who is also a co-author of the article.*

The construct validity of the questionnaire was assessed by an exploratory factor analysis to determine whether similar statements measured the same latent variable as proposed by several authors (22-23). The set of statements in the first part of the questionnaire is designed to measure elements of the work environment based on the practice environment scale of the nursing work index (PES-NWI) (8). All items were measured on a four-point Likert agreement scale. Four factors were extracted using the principal axis factoring method and orthogonal rotation, namely: interpersonal relationships and teamwork (16.4% of variance explained), nurses' co-decision-making and development prospects(10.3% of variance explained), organisational priorities regarding the quality of patient care (9.1% of variance explained) and support of the nursing management (8.9% of variance explained). The first factor, interpersonal relationships and teamwork, included seven items. An exemplary item is “Doctors respect nurses as professionals.” The second factor, nurses’ co-decision-making and development prospects included five items with an exemplary item being “Registered nurses participate in the hospital’s internal management”. The third factor, organisational priorities regarding the quality of care included five items. The item with the highest factor weight is “There is an active quality assurance program”. The fourth factor, organisational priorities regarding support of the nursing management included five items. An exemplary item is “The supervising nurse manages and leads the department well”. One item “There are written, up-to-date nursing care plans for all patients.“ had high factor weights on two factors, and seven items did not have high factor weight on any of the factors. These items were excluded from the calculation of composite score (an average of items with high weights on a single factor). Cronbach’s alpha coefficient indicated good measurement reliability for all factors (range 0.74 to 0.92) (S1 Table).

In statements about patient safety culture (measured on the five-point Likert agreement scale), two latent variables (factors) were extracted using factor analysis, namely: mutual trust (23.9% of variance explained) and the importance of patient safety and feedback (19.7% of variance explained). Mutual trust included four items, which were reverse coded for the analysis. An exemplary item (reversed coded for the analysis) is “When changing shifts important information about patient care is often lost”. The importance of safety included two items, which were reverse coded for the analysis. An exemplary item is “The hospital management’s actions show that patient safety is one of the most important tasks”. One item did not have a high factor weight on any of the factors and was excluded from the calculation of the composite score (an average of items with high weights on each factor). In this case, Cronbach’s alpha coefficient also demonstrated good reliability of the measurement (0.71 and 0.72) (S2 Table).

**Supporting information**

S1 Table: Factor weights (>0.40) obtained by factor analysis (principal axis factoring method and orthogonal rotation) on statements regarding work environment.

|  | **Factor weights** | | | |
| --- | --- | --- | --- | --- |
|  | **F1** | **F2** | **F3** | **F4** |
| A_1_14 Work performed well is praised and acknowledged. |  |  |  | 0.44 |
| A_1_11.The hospital’s head nurse is very visible and accessible to the staff. |  |  |  | 0.50 |
| A_1_23 The management listens to employees’ problems and responds to them. |  |  |  | 0.51 |
| A_1_22 The department’s supervising nurse supports nurses in their decisions, even when they disagree with the doctor. |  |  |  | 0.54 |
| A_1_10 The supervising nurse manages and leads the department well. |  |  |  | 0.76 |
| A_1_31 There are written, up-to-date nursing care plans for all patients. |  | 0.45 | 0.40 |  |
| A_1_32 Assigning patient care tasks promotes consistent nursing care (i.e., a patient is cared for by the same nurse day after day). |  |  | 0.41 |  |
| A_1_27 There is a mentoring program for newly hired nurses. |  |  | 0.41 |  |
| A_1_12. There is enough staff for the work to get done. |  |  | 0.54 |  |
| A_1_19 There is a clear philosophy of nursing care in the area of patient care. |  |  | 0.59 |  |
| A_1_24. There is an active quality assurance program. |  |  | 0.64 |  |
| A_1_28 Patient care is based on a nursing care model and not a medical model. |  | 0.54 |  |  |
| A_1_4 Active employee development programs or ongoing training for nurses are available. |  | 0.56 |  |  |
| A_1_6 Registered nurses have the opportunity to participate in preparing nursing care instructions. |  | 0.57 |  |  |
| A_1_29. Registered nurses are able to be members of hospital committees and nursing care committees. |  | 0.59 |  |  |
| A_1_25 Registered nurses participate in the hospital’s internal management (e.g., on professional committees). |  | 0.63 |  |  |
| A_1_3 My superiors support nurses. | 0.54 |  |  |  |
| A_1_17 There is a lot of teamwork between nurses and doctors. | 0.67 |  |  |  |
| A_1_30 Doctors value nurses highly. | 0.72 |  |  |  |
| A_1_2 Working relationships between doctors and nurses are good. | 0.72 |  |  |  |
| A_1_13 Doctors recognize nurses’ contributions to patient care. | 0.73 |  |  |  |
| A_1_26 Nurses and doctors work well together. | 0.74 |  |  |  |
| A_1_21 Doctors respect nurses as professionals. | 0.80 |  |  |  |
| A_1_1 Suitable support services enable me to dedicate time to my patients. |  |  |  |  |
| A_1_8 I have enough time and opportunity to talk with other nurses about problems with patient care. |  |  |  |  |
| A_1_9 There are enough registered nurses on the staff for quality patient care. |  |  |  |  |
| A_1_15 The management expects high standards of nursing care. |  |  |  |  |
| A_1_16 The hospital’s head nurse has equal authority and authorization as the hospital’s top management staff. |  |  |  |  |
| A_1_18 There are opportunities for advancement. |  |  |  |  |
| A_1_20 I work with nurses that are clinically trained. |  |  |  |  |
|  |  |  |  |  |
| **The share of explained variance** | **16.4** | **10.3** | **9.1** | **8.9** |
| **Cronbach's alpha coefficient** | **0.92** | **0.82** | **0.74** | **0.76** |

**S1 Table legend:**

**F1:** Interpersonal relationships and teamwork

**F2:** Nurses' co-decision-making and development prospects

**F3:** Organisational priorities regarding the quality of patient care

**F4:** Support of the nursing management

S2 Table: Factor weights (>0.40) obtained by factor analysis (principal axis factoring method and orthogonal rotation) on statements regarding patient safety culture.

| **Rotated Factor Matrix^a^** | **Distrust, poor communication**  **(F1)** | **Lack of safety culture (F2)** |
| --- | --- | --- |
|  | **Factor weights** | |
|  | **Mutual trust** | **The importance of safety culture** |
| It seems to staff that others resent their mistakes.(R) | 0.40 |  |
| When changing shifts important information about patient care is often lost.(R) | 0.83 |  |
| When moving from one unit to another things are “poorly handed over”.(R) | 0.65 |  |
| The staff openly express doubts about their superiors’ decisions and actions.(R) | 0.57 |  |
| In our unit we talk about how to make sure not to repeat mistakes. |  |  |
| We receive feedback on changes made in response to reports on events. |  | 0.72 |
| The hospital management’s actions show that patient safety is one of the most important tasks. |  | 0.77 |
| **The share of explained variance** | **23.9** | **19.7** |
| **Cronbach's alpha coefficient** | **0.72** | **0.71** |

**S2 Table legend :**

**F1**: Mutual trust

**F2**: The importance of patient safety and feedback

**The methods section should define all the key outcome variables and how these were measured. For example, it does not state that adverse events will be collected. The methods section should also describe exactly how patient safety and quality of nursing care were measured, as it is not clear for the reader. Methods should also explain how job satisfaction was measured as it is mentioned first on line 193. It is not clear how the sample size was determined.**

*Dear reviewer, thank you for your valuable comments. Please find in manuscript in Section Methods/ Statistical analysis inclusions of our answer, as shown below (underlined). We removed the statement of job satisfaction.*

In the study, we used the original questionnaire with all-encompassing scales, although only results of the part of the questionnaire are presented in this paper. The mean number of patients per nurse during their last shift was calculated based on self-assessment provided by nurses. Items relating to nurses’ work environment, safety culture, overall assessment of nurses’ work environment (a single item), overall assessment of patient safety in the department (a single item), overall quality of nursing care in the department (a single item) and adverse events were described by percentages. Composite scores per dimension of nurses’ work environment and patient safety culture (as evaluated by factor analysis) were calculated and used in multiple linear regression analysis. Two multiple regression models were built to examine the relationship between nurses’ work environment and patient safety culture. Four dimensions of nurses’ work environment were included as independent variables and each dimension of patient safety culture (mutual trust, importance of safety culture) was included a dependent variable in the regression model. Comparison between nurses with and without bachelor’s degree in the overall assessment of the work environment was done by Mann-Whitney U test. Spearman correlation coefficient was calculated between each dimension of patient safety culture or nurses’ work environment and frequency of each adverse event. Statistical testing was performed at the 0.05 significance level. No correction for multiple testing was applied. Statistical analysis was performed using SPSS software, v. 26.

No a priori sample size was determined, however post-hoc sample size calculation indicates that for achieving 80 % power of the multiple linear regression model with four predictors at significance level α equal to 0.05 and effect size as determined by R^2^ equal to 0.17, the sample size of 85 would suffice.

*Dear reviewer, please find in the manuscript in Section Methods/* *Sampling, inclusion criteria and sample description the comparison of the structure of the sample of the sample to the population, as shown below (underlined). We have to say that the other reviewer also found the same issue to be discussed as you, so with this reply we answer to both of you.*

Simple random sampling of six out of ten Departments of the Division of Internal Medicine and from the nine Departments of the Division of Surgery at the UMC Ljubljana was performed using the “Simple Random Sampling Applet” computer programme (24). Randomization was done at the department level. This is one of the probability sampling methods that yields a representative sample if the response is large and the non-response is by the chance. Of the 384 nursing staff in the selected departments, 270 nurses (70%) were present at the workplace at the time of the survey. The questionnaire was sent to all present nurses in the selected departments and 147 questionnaires (54%) were completed and returned. The majority (90.5%) of the participants were female and full-time employees (94.6%). Their average (standard deviation) age was 40 (SD=10.9) years, and 63.3% of the nurses had a bachelor*’*s degree. A comparison of the structure of the sample, according to the level of nurses’ education, shows (Table 1) that the structure of the sample statistically significantly differs from that in the population (p < 0.001). The nursers with bachelor’s degrees are overrepresented in the sample. The gender structure, however, does not statistically significantly differ from that in the population (p = 0.063). In our study, the calculated ratio was 9.94 patients per nurse per work shift. We obtained this ratio based on the data from the question: “How many patients were you directly responsible for during your most recent job?” All forms of work shifts were included.

Table 1: Sample description.

|  | Sample  (n = 147) | Population (n = 1393) | P |
| --- | --- | --- | --- |
| Female sex f (%) | 133 (90.5) | 1184 (85) | 0.063 |
| Mean (SD) age in years | 40 (10.9) | - |  |
| Bachelor's degree | 93 (63.3) | 600 (43.1) | < 0.001 |

**I found the result section difficult to understand without referring to the original RN4CAST questionnaire. It needs to be clearer for the reader to understand all the variables being measured and presented. Many of the measures appear to have come from single questions on the RN4CAST (A3, B4 and B1) which could be made clearer to the reader. If possible, the authors should present effect sizes/absolute values as well as p values for correlation, as it is difficult to judge whether findings would be clinically important.**

*Dear reviewer, thank you for your comments. Please find in the manuscript in Sections Results and Supporting information inclusions of our answers, as shown below.*

Nurses gave the highest rating to the statements that “management expects high standards of nursing care” (47.6% completely agree and 41.5% agree responses) and that “they work with clinically qualified nurses” (43.5% completely agree and 44.9% agree responses). They gave the lowest rating to the statements that “assigning patient care tasks promotes continuity of care” (24.5% completely disagree and 29.9% disagree responses) and that “there is enough staff to get the job done” (35.4% completely disagree and 33.3% disagree responses).

A comparison of the percentage of nurses' responses regarding the general assessment of the work environment, patient safety and the quality of nursing care is summarized in Figure 1.

*Figure 1: Evaluation responses regarding the overall assessment of the work environment in the department, patient safety in the department and the quality of nursing care (N=147).*

A3 – How would you rate the working conditions in your current job at this hospital (e.g., suitable equipment, relations with coworkers, support from superiors); B1 - How would you rate the overall quality of nursing care that patients receive in your department/unit; B4 - Rate the overall safety of patients in your department/unit.

From the statements measuring the patient safety culture, nurses agreed and most with the statement that “in the department, they work on preventing mistakes being repeated” (68.5% agree and strongly agree responses). The agreement with the reversely coded statement that “others resent their mistakes” was the lowest (34% agree and strongly agree responses) (Figure 2). The lowest share of strongly agree responses was regarding “the receiving of feedback on changes made in response to report events”.

*Figure 2: Patient safety culture by statements (n = 147).*

Among the 8 quality indicators of healthcare listed in the questionnaire as adverse events occurring to patients or employees, verbal abuse of nurses by patients and/or their relatives as well as by staff stood out. About one-fifth (19%) of the surveyed nurses perceived weekly verbal abuse by patients and/or their relatives. The share of responses confirming the occurrence of verbal abuse of nurses by patients or their relatives once to several times a month was even higher (44.9%). The occurrence of verbal abuse of nurses by staff was perceived by 12.2% of the respondents on a weekly basis, and once to several times a month by 35.4% of nurses.

The incidence of pressure sores after admission once or more per month was confirmed by nurses by 29.9%. Similarly, complaints of patients or their relatives were detected among nurses once or more per month by 29.3%. Work-related physical injuries occurred to nurses once or more per month in 20.4%. (Figure 3).

*Figure 3:Frequency of adverse events occurring to patients or staff (quality indicators of healthcare) (n=147)*

**3.1 Determining the relationship between the perception of the work environment and safety culture and the quality of patient care**

Using multiple linear regression with the four dimensions of nurses’ work environment as independent and mutual trust as one of the two dimensions of patient safety culture as dependent variable (Table 2). We found that two dimensions of nurses’ work environment were related to mutual trust, namely organisational priorities regarding the quality of care (B = 0.30; p = 0.016) and management support of nursing care (B = 0.35; p = 0.003). Both regression coefficients are positive indicating that higher organisational priorities regarding the quality of patient care and higher management support of nursing care are reflected in higher mutual trust between healthcare staff.

*Table 2: Relationship between perception of the dimensions of the work environment and the mutual trust, the first dimension of the patient safety culture (result of multiple linear regression).*

|  | Regression coefficient | P |
| --- | --- | --- |
| Intercept | 1.78 | <0.001 |
| Interpersonal relationships and teamwork | -0.06 | 0.609 |
| Nurses’ co-decision-making and the opportunity for development | -0.05 | 0.693 |
| Organisational priorities regarding the quality of patient care | 0.30 | **0.016** |
| Management support nursing care | 0.35 | **0.003** |

R^2^=0.17

The second multiple regression model included the four dimensions of nurses’ work environment as independent and the second dimension of patient safety culture, namely the importance of patient safety, as dependent variable (Table 3). Results indicate that the importance of patient safety is positively related to the nurses’ co-decision-making and the opportunity for development (B = 0.31; p = 0.048) and with organisational priorities regarding the quality of patient care (B = 0.49; p = 0.001). Higher importance of patient safety is present when nurses have the opportunity for professional development and cooperate in decision making. The importance of patient safety goes hand in hand with firmly set priorities regarding the quality of patient care.

*Table 3: Relationship between perception of the dimensions of the work environment and the importance of patient safety, the second dimension of the patient safety culture (results of multiple linear regression).*

|  | Regression coefficient | P |
| --- | --- | --- |
| Intercept | 1.05 | 0.006 |
| Interpersonal relationships and teamwork | -0.01 | 0.939 |
| Nurses’ co-decision-making and the opportunity for development | 0.31 | **0.048** |
| Organisational priorities regarding the quality of patient care | 0.49 | **0.001** |
| Management support nursing care | -0.02 | 0.907 |

R^2^=0.20

We found a positive correlation between all dimensions of the work environment and nurses' certainty that patients can care for themselves at discharge (S3 Table). We also confirmed a positive correlation between most dimensions of the work environment and the general assessment of the quality of nursing care in the department as well as the overall assessment of the quality of hospital care of patients in the past year (S3 Table).

Adverse events involving patients or nurses showed a negative correlation with at least two of the dimensions of the nurses’ perception of the work environment (Table 4). The correlation coefficients, however, indicate a weak relationship between variables. The highest, but still moderate, negative correlation exists between verbal abuse of nurses by staff and management support of nursing care (r = 0.38; p < 0.001) and the former and overall assessment of the work environment in the hospital (r = - 0.37; p < 0.001).

*Table 4: Spearman's correlation coefficient between adverse events and perception of the work environment.*

|  | Pressure ulcers after admission | | Patients' falls leading to injuries | | Healthcare- associated urinary tract infections | | Verbal abuse of nurses by patients and/or their families | | Verbal abuse of nurses by staff | |
| --- | --- | --- | --- | --- | --- | --- | --- | --- | --- | --- |
|  | r P | | r P | | r P | | r P | | r P | |
| Interpersonal relationships and teamwork | -0.24 | **0.003** | -0.18 | **0.031** | -0.21 | 0.009 | -0.07 | 0.423 | -0.11 | 0.192 |
| Nurses'  co-decision-making and  the opportunity for development | -0.19 | **0.024** | -0.24 | **0.004** | -0.17 | **0.042** | -0.25 | **0.003** | -0.30 | **<0.001** |
| Organisational priorities according to quality of care | -0.11 | 0.173 | -0.23 | **0.005** | -0.19 | **0.023** | -0.20 | **0.018** | -0.25 | **0.002** |
| Management supports nursing care | -0.04 | 0.654 | -0.07 | 0.417 | -0.15 | 0.070 | -0.25 | **0.002** | -0.38 | **<0.001** |
| Overall assessment of the work environment in the hospital | -0.14 | 0.097 | -0.16 | 0.051 | -0.21 | **0.010** | -0.33 | **<0.001** | -0.37 | **<0.001** |

r = Spearman's correlation coefficient

**3.****2 Perception of the work environment and nurses' education**

We then investigated whether there is a difference in the perception of the work environment according to nurses' acquired education (with a bachelor*’*s degree/ without a bachelor*’*s degree). The study involved 63.3 % graduate nurses (at the time of the study, their proportion at the hospital’s departments was 43.1%). Nurses with a bachelor*’*s degree gave a lower overall assessment of the work environment (U-value=1821.5, p=0,003). Also, nurses without a bachelor’s degree rated the dimension organizational priorities regarding the quality of patient care more positively (M=2.59, SD=0.56) than nurses with bachelor’s degree (M=2.33, SD=0.66, p=0.015).

In Slovenia, nursing education takes place in two stages, in accordance with the standards of the European Directive 2013/55/EU. The professional competencies of the graduate nurse as a healthcare provider in the healthcare team comply with the competencies of the above-mentioned directive after completing 3-years of the first cycle of Bologna higher education. After completing four years of education, the healthcare technician is qualified to observe and monitor the patient's state of health, perform tasks as directed by a graduate nurse or medical doctor, report in the team, assist in patient activities of daily living, provide first aid, and care for the dying and deceased. His/her professional competencies enable him/her to carry out part of the nursing activities and diagnostic-therapeutic program independently, and part as a co-worker in a team led by a graduate nurse (25).

**Supporting information**

*S3 Table: Spearman's correlation coefficient between the general assessment of the quality of care, the assessment of self-care and the assessment of the quality of care in the previous year and the perception of the work environment*

|  |  | Interpersonal relationships and teamwork | Nurses’ co-decision-making and the opportunity for development | Organisational priorities according to the quality of care | Management supports nursing care | Assessment of the work environment in your workplace in the hospital |
| --- | --- | --- | --- | --- | --- | --- |
| How would you generally assess the quality of nursing care on your ward? | r | 0.12 | 0.25 | 0.32 | 0.27 | 0.32 |
|  | P | 0.160 | **0.002** | **< 0.001** | **0.001** | **< 0.001** |
| How certain are you that your patients can care for themselves after discharge? | r | 0.20 | 0.36 | 0.39 | 0.24 | 0.29 |
|  | P | **0.015** | **< 0.001** | **< 0.001** | **0.004** | **< 0.001** |
| In the past year, the quality of patient care in your hospital... | r | 0.12 | 0.29 | 0.30 | 0.34 | 0.27 |
|  | P | 0.138 | **< 0.001** | **< 0.001** | **< 0.001** | **0.001** |

**The discussion should have a section on study limitations and potential sources of bias. The discussion relates well to previous literature however the conclusion on line 243** **that poor patient safety arises from a poor working environment is not substantiated from the data. The direction of effect has not been ascertained as this is a cross sectional study. The generalisability of findings could have been considered.**

*Dear reviewer, than you for your comments. Please find in the manuscript in Sections Discussion inclusions of our answers, as shown below (underlined).*

1. *We added* *Strengths and limitations – please find it in manustript in Section Discussion, ass shown below.*

The limitation of the research is that no correction was made for multiple testing, hence some false positive statistically significant associations might have been discovered. For more rigorous interpretation of results only those with p < 0.001 could be interpreted as statistically significant.

Respondents' perceptions expressed their view on probabilities and predictions, which may differ from actual situations. Further research would be needed to clarify the more exposed results of our research.

As mentioned earlier, we conducted the research to investigate the relationship between the perception of the nurses’ work environment and selected variables. The innovative European RN4CAST project is still relevant. It is important to research and discern how to create a positive work environment for nurses and other staff, that impacts sick leave and the recruitment and retention rates. Our research was carried out ten years after the European project, which may be a partial limitation.The data presented in the introduction confirm the deterioration of the situation in Slovenian nursing care. No decisive or substantive changes have been made in the past ten years, nor systematic improvements that could limit the conclusions of direct comparison of our results with the European project. However, in some cases, our results were more comparable to those of individual countries than to the overall average.

Our study is a good starting point for a wider study in all Slovenian hospitals to identify and predict the needs for nursing care providers in light of the current situation and all characteristics of nurses' work environment.

2. *Dear reviewer, we corrected the statement »that poor patient safety arises from a poor working environment« in Section Discussion, as shown below.*

In our study. we demonstrated a correlation between the assessment of nurses’ perception of the work environment and the overall assessment of patient safety. Almost one-third of the respondents rated patient safety as poor or satisfactory. Similarly, the results of a survey in twelve Slovenian hospitals in 2013 showed an overall low patient safety culture. The following elements of the safety culture were rated the worst: teamwork across hospital units, non-punitive response to errors, hospital management support for patient safety, and staffing (31). Foreign researchers have reached conclusions, that a better hospital work environment for nurses increases the chance of survival of patients with cardiac arrest by 16% (32). In another study, it was shown that there is an 11% increased chance of 30-day survival in elderly patients on mechanical ventilation in better work environments, and if more graduate nurses are present (33). Our respondents rated the work environment as poor or satisfactorly in almost half of answers (48.9%).

**The conclusion is succinct and based on the evidence presented.**

**Overall, I believe that the research has been conducted in a rigorous way using a defined population and it adds to new knowledge. However, the paper itself could be improved so that the methods and results sections are more clearly linked. The introduction and discussion sections could be strengthened as highlighted above.**

*Dear reviewer, than you for your valuable and encouraging comments.*

*As presented in the first answer, we have supplemented the Section Introduction with the necessary information. Text has also been added to the Section Discussion, as shown below (underlined).*

In this study, we used the RN4CAST questionnaire to determine the correlation between nurses' perception of the work environment and patient safety and the quality of nursing care. Our findings showed that two-third of nurses (68%) rated the patient safety culture as “good” or “excellent”. The result is surprising, as it is 24 percentage points lower than the European average (92%) (5). The evidence from a European survey supports the fact that patient safety is a key indicator of the quality of medical treatment and nursing care (5,10,12).

Various U.S. researchers have shown that a poorer safety culture of the hospital increased the 30-day mortality rate among patients with acute myocardial infarction (26) and that in patients with heart failure, the possibility of readmission within 30 days after discharge from hospital increased by 2-8% if certain nursing care activities were not performed (27). Nursing activities left undone can pose a high risk to quality and safe patient care (28).

Among the statements about the patient safety culture, the lowest-rated statement that “employees receive feedback on changes made in response to reports on events” was alarming. To eliminate the shortcomings of existing work systems and processes, it is important that reporting and analysis of adverse events allow for timely corrective action (29) while considering interprofessional differences in perception of patient safety culture as well as certain influential factors (sufficient staff, lack of time) (30).

In our study. we demonstrated a correlation between the assessment of nurses’ perception of the work environment and the overall assessment of patient safety. Almost one-third of the respondents rated patient safety as poor or satisfactory. Similarly, the results of a survey in twelve Slovenian hospitals in 2013 showed an overall low patient safety culture. The following elements of the safety culture were rated the worst: teamwork across hospital units, non-punitive response to errors, hospital management support for patient safety, and staffing (31). Foreign researchers have reached conclusions, that a better hospital work environment for nurses increases the chance of survival of patients with cardiac arrest by 16% (32). In another study, it was shown that there is an 11% increased chance of 30-day survival in elderly patients on mechanical ventilation in better work environments, and if more graduate nurses are present (33). Our respondents rated the work environment as poor or satisfactorly in almost half of answers (48.9%).

The general assessment of the work environment showed that half of the nurses (49%) assessed the work environment as “poor” or “satisfactory”. The result of the assessment of the work environment correlated with the percentage of nurses who would leave their current job in the next year due to dissatisfaction with the work environment (51%). Sermeus et al. (8) found that nurses in all participating countries had a negative perception of their work environment and expressed their intention to leave their current job in one year, the percentage varying from country to country (20% to 50%), with an average of 35.6% (9). When looking for measures to retain nurses, the major Slovenian hospital identified the factors influencing nurses’ leaving: inadequate pay (for less work in nursing or for easier work outside of nursing), lack of professional affiliation and the futility of performing a demanding profession in nursing care, poor interpersonal relationships and communication in health care, inadequate mentoring, desire to work in another field, insufficient staff, the rapid development of the profession and innovations, physical demands (34). The increased turnover of nursing staff is a global and long-standing social problem, the causes of which are multifaceted, often similar and comparable regardless of the diversity of the geographical and cultural environment.

Compared to the overall assessment of patient safety, the quality of nursing care in the hospital was rated high in the study. The individual dimensions of the work environment, as well as its general assessment, showed a positive correlation with the general assessment of the quality of nursing care, with the nurses' confidence in patient self-care after discharge, and with a better assessment of the quality of patient care in the last year.

Quality indicators of healthcare as adverse events that occurred to patients or staff confirmed a negative correlation with nurses’ perception of the work environment. Some associate the occurrence of adverse events with poor communication and poor cooperation in interdisciplinary teams, resulting in inappropriate handover protocols and a lack of recognition of and respect for the role of the individual (35), while at the same time attributing an important role to the leaders of nursing care teams (36). A poor work environment as well as a high ratio of patient to nurse certainly increases the possibility of adverse events (37, 38). The high ratio of the number of patients per nurse calculated in our study corresponds with the lowest assessment of agreement of the participating nurses that “there are not enough staff on the department to get the job done”. According to European researchers (8), the number of nurses in individual countries is not a true indicator of the adequacy of the number of nursing staff. When there is a shortage of nursing staff, employers strive to make the most efficient use of the workforce, but research shows the correlation between long shifts or overtime work and nursing activities not carried out, which results in a poorer quality of care (11-12). The recent study in Slovenian hospitals also confirms the workload as the most stressful factor among nurses, which consequently reduces the quality of care (39). Objectively measured personnel data based on the Slovenian categorization of the intensity of hospital nursing also prove the correlation of the long-term shortage of nursing staff with some national quality indicators of healthcare (40).

The most pronounced negative impact on the quality of nursing care in the study was the perceived verbal abuse of nurses by patients and/or their relatives on a monthly basis (44.9%), which was almost twice as high as the average in the European study (26%), and abuse by staff (35.4%), which was four times higher than the European average (8%) (13). Unstable conditions in the work environment (insufficient number of graduate nurses, increased workloads, reduced support from management, poor team relations, etc.) have been shown to promote abuse of nurses and to have a greater impact than the patient population itself (41). We have previously presented the findings of our study on the poor assessment of the work environment by almost half of the respondents. In 2018, a survey was conducted in three Slovenian healthcare institutions in various places on the prevalence of violence among healthcare employees. Employees expressed the opinion that they had experienced verbal violence from patients or their relatives more than ten times in one year in 24.1% of cases, while 10% confirmed they had experienced violence from co-workers or superiors (42). A previous Slovenian survey among nurses on workplace violence revealed that the most frequent perpetrators of verbal abuse were patients (39.3% of respondents) and co-workers (39.6% of respondents) (43).

European research has confirmed, among other things, that the level of nurses’ education is important for reducing adverse events (10). In our study, regarding the link between nurses’ level of education and their perception of the work environment, we established a worse, negative general assessment of the work environment as well as a poorer assessment of dimension of the work environment on organizational priorities regarding the quality of care among graduate nurses. The percentage of graduate nurses who expressed a poor assessment of the work environment was almost one-third higher than healthcare technicians. Certainly, acquired knowledge, experience and professional competencies encourage responsible judgment, decision support, and high-level practice within the framework of nursing care regarding safe and quality patient care, as well as enabling critical evaluation of individual events and situations (7,44). In our study, nurses expressed a desire for greater educational opportunities as well as more prospects for advancement. On the other hand, 57% of respondents thought that there are enough graduate nurses on the department for quality patient care (answers “I partially agree” and “strongly agree”), which is contrary to the results of the European study, where, on average, almost 70% of the respondents expressed an opinion on the shortage of graduate nurses on the department (10).
