# Supplementary material for: The relationship between the nurses’ work environment and the quality and safe nursing care: Slovenian study using the RN4CAST questionnaire
Source: PLoS One. 2021 Dec 20;16(12):e0261466. doi: 10.1371/journal.pone.0261466 (PMC8687596; doi:10.1371/journal.pone.0261466)
Supplement: Attachment — Submitted filename: Response to reviewer 2.docx [file pone.0261466.s007.docx]

**The relationship between the nurses' work environment and the quality and safe nursing care: Slovenian study using the RN4CAST questionnaire**

*Dear reviewer,*

*thank you for evaluating the revised manuscript. We followed your comments and made all necessary corrections and changes according to your suggestions. These changes are shown below.*

*We wish you successful work in the future as well.*

**I would suggest moving the last paragraph of the introduction to the methods section, as it describes the setting and the approvals obtained to conduct the study (e.g. could be moved to ‘study and data analysis’).**

*Dear reviewer, we have considered your suggestion and move the last paragraph of the Introduction to the section Study and data analysis, as shown below (underlined).*

**Study and data analysis**

Consent to use the questionnaire for nurses from European research was obtained in August 2018 by the European coordinator of the EU-FP7 RN4CAST-project, Full Professor Walter Sermeus, provided that we conduct a study in one hospital. Therefore we decided to conduct a study among graduate nurses and healthcare technicians at the UMC Ljubljana. In early 2019, 1721 nurses were employed in hospital departments of UMC Ljubljana, of which 47.3% were nurses with a bachelor*’*s degree (24). Ethical approval for the pilot study was granted by the Committee for Medical Ethics of the Republic of Slovenia (Reference no. KME RS.0120-490/2018/5). Before the start of the research work, we addressed a request for a pilot study to the head nurse of UMC Ljubljana. We also previously obtained consent for research from the hospital research group in nursing and all other necessary permits (Leadership team of the Division of Internal Medicine, Leadership team of the Division of Surgery).

The study was conducted from January 16th, 2019, to March 1st, 2019. We previously notified the head nurses and heads of selected departments by e-mail of their random selection and requested their cooperation. We presented the purpose of the study and how it would be conducted to all participating nurses in the selected departments in advance and informed them of the voluntary basis of their decision and the anonymity of participation. The survey questionnaires were handed out to participants in a sealed envelope, and a new envelope with the title of the submission was enclosed for its return. Envelopes with the completed questionnaires were sent daily from the administrative offices of individual departments with the rest of the internal mail to the one selected administrative office at the Division of Surgery. The time limit for completing and returning the questionnaires was a maximum of three weeks.

**Similarly, some of the findings were presented in the methods section. I would suggest moving lines 144-155 to the results section.**

*Dear reviewer, we moved the proposed text (findings) to the section Results, while merging the sub-sections Sampling, inclusion and exclusion criteria, as shown below. One sentence from section Introduction is also added.*

**Sampling, inclusion and exclusion criteria**

University Medical Centre Ljubljana (UMC Ljubljana) is the largest Slovenian hospital with 2150 beds. Simple random sampling of six out of ten Departments of the Division of Internal Medicine and from the nine Departments of the Division of Surgery at the UMC Ljubljana was performed using the “Simple Random Sampling Applet” computer programme (23). Randomization was done at the department level. This is one of the probability sampling methods that yields a representative sample if the response is large and the non-response is by the chance.

By following the RN4CAST methodology and work protocol, only adult medical-surgical care nursing units were included in the study. The data of different elements of nursing practice environment to patient safety and clinical outcomes are best researched and documented within these work domains (20), thus enabling a more reliable comparison. Specialized nursing units, such as the Departments of Paediatrics, Intensive Care Units, Long-Term Care Units, Emergency Departments, Department of Anaesthetics Care and Operating Theatres were not included in the selection process of our research.

# **Results**

Of the 384 nursing staff in the selected departments, 270 nurses (70%) were present at the workplace at the time of the survey. The questionnaire was sent to all present nurses in the selected departments and 147 questionnaires (54%) were completed and returned. The majority (90.5%) of the participants were female and full-time employees (94.6%). Their average (standard deviation) age was 40 (SD=10.9) years, and 63.3% of the nurses had a bachelor*’*s degree. A comparison of the structure of the sample, according to the level of nurses’ education, shows (Table 1) that the structure of the sample statistically significantly differs from that in the population (p < 0.001). The nursers with bachelor’s degrees are overrepresented in the sample. The gender structure, however, does not statistically significantly differ from that in the population (p = 0.063). In our study, the calculated ratio was 9.94 patients per nurse per work shift. We obtained this ratio based on the data from the question: “How many patients were you directly responsible for during your most recent job?” All forms of work shifts were included.

Table 1: Sample description.

|  | Sample  (n = 147) | Population (n = 1393) | P |
| --- | --- | --- | --- |
| Female sex f (%) | 133 (90.5) | 1184 (85) | 0.063 |
| Mean (SD) age in years | 40 (10.9) | - |  |
| Bachelor's degree | 93 (63.3) | 600 (43.1) | < 0.001 |

**Line 216 – Should this be “From the statements measuring the patient safety culture, nurses agreed most with the statement (…)”?

Lines 217 and 219 – When you say “68.5% agree and strongly agree responses” do you mean that 68.5% agree or strongly agree with the statement? Same for line 219. This is what I gather from figure 2, please reword if correct.**
*Dear reviewer, we provide a common answer to these two proposed corrections (underlined).*

From the statements measuring the patient safety culture, nurses agreed and most with the statement “in the department, they work on preventing mistakes being repeated” (68.5% of them agreed or strongly agreed with the statement). The agreement with the reversely coded statement that “others resent their mistakes” was the lowest (34% of them agreed or strongly agreed with the statement) (Figure 2). The lowest share of strongly agree responses was regarding “the receiving of feedback on changes made in response to report events”.

*Due to the correct interpretation of the results, we similarly reworded the sentences (lines 201-207) in the previous paragraph, as shown below (underlined).*

Nurses gave the highest rating to the statements that “management expects high standards of nursing care” (47.6% of respondents completely agreed and 41.5% of them agreed with the statement) and that “they work with clinically qualified nurses” (43.5% of respondents completely agreed and 44.9% of them agreed with the statement). They gave the lowest rating to the statements that “assigning patient care tasks promotes continuity of care” (24.5% of respondents completely disagreed and 29.9% of them disagreed with the statement) and that “there is enough staff to get the job done” (35.4% of respondents completely disagreed and 33.3% of them disagreed with the statement) (S1 Figure).

*Dear reviewer, we would like to inform you that we added S1 Figure at the suggestion of the another reviewer in the Supporting information, where the statements relating to the perception of the work environment in the arithmetic mean and standard deviation are presented.*

*S1 Figure****:*** *Average degree of agreement (M – arithmetic mean) and standard deviation (SD) for statements relating to the perception of the work environment.*

**Lines 282-284 – I would suggest spelling out M and SD.**

*Dear reviewer, we have removed statistical values from the sentence, as shown below. The results of the statement are presented from the S4 Table, which was added to Supporting information (proposal of another reviewer).*

Also nurses without a bachelor’s degree rated the dimension organizational priorities regarding the quality of patient care more positively than nurses with bachelor’s degrees (S4 Table).

*S4 Table: Perception of the work environment and nurses' education*

|  | A bachelor’s degree | M | SD | P |
| --- | --- | --- | --- | --- |
| **Interpersonal relationships and teamwork** | Yes | 2.45 | 0.69 | 0.562 |
|  | No | 2.51 | 0.60 |  |
| **Nurses’ co-decision-making and the opportunity for development** | Yes | 2.52 | 0.66 | 0.143 |
|  | No | 2.66 | 0.47 |  |
| **Organisational priorities regarding the quality of patient care** | Yes | **2.33** | **0.66** | **0.015** |
|  | No | **2.59** | **0.56** |  |
| **Management support nursing care** | Yes | 2.55 | 0.69 | 0.72 |
|  | No | 2.51 | 0.59 |  |

*Dear reviewer, we have also considered your next corrections, as shown below. Thank you again for all your valuable help and expert suggestions.*

**Line 312 – Should contain a comma instead of a full stop i.e. “In our study, we demonstrated (…)”.**
In our study, we demonstrated a correlation between the assessment of nurses’ perception of the work environment and the overall assessment of patient safety.

**Line 217 – Delete comma “Foreign researchers have reached conclusions,”**

Foreign researchers have reached conclusions that a better hospital work environment for nurses increases the chance of survival of patients with cardiac arrest by 16% (32). **Lines 323-324 seem to repeat the same information reported in lines 321-322. If so, I would suggest deleting lines 321-322.**

… In another study, it was shown that there is an 11% increased chance of 30-day survival in elderly patients on mechanical ventilation in better work environments, and if more graduate nurses are present (33).

The general assessment of the work environment showed that half of the nurses (49%) assessed the work environment as “poor” or “satisfactory”.
